# A two-faced selectivity solution to target SMARCA2 for cancer therapy

**DOI:** 10.1038/s41467-023-36238-0

**Published:** 2023-01-31

**Authors:** John D. Harling, Christopher P. Tinworth

**Affiliations:** Dept. of Medicinal Chemistry, GSK, Gunnels Wood Road, Stevenage, SG1 2NY UK

**Keywords:** Cancer therapy, Cancer, Target validation

## Abstract

Two new studies exploring PROTAC-mediated degradation of SMARCA2 for cancer therapy solve an apparently intractable selectivity challenge with SMARCA4 by utilising the requirement for a productive ternary complex between the protein, PROTAC and ligase complex.

Achieving selectivity with small-molecule drugs is often one of the sternest challenges faced by those involved in drug discovery and countless promising programs have ultimately failed due to the inability to adequately distinguish between highly homologous binding sites in related proteins. One such example exists with the multi-subunit switch/sucrose nonfermentable (SWI/SNF or BAF) complex which enables chromatin remodelling to control key cellular processes^1^. This complex contains either the SMARCA2 or SMARCA4 helicase^2^, and loss of function mutations in SMARCA4 are a hallmark of multiple cancers^3^. Functional genomic studies have revealed a synthetically lethal pairing between the inactivated SMARCA4 and wild-type SMARCA2, driven by ATPase domain activity, but not by the bromodomain found in the same protein^4^. Dual inhibitors of the SMARCA2 and SMARCA4 ATPase domain are poorly tolerated^5^, so while selectively targeting SMARCA2 represents an attractive strategy to overcome this, it has not proved possible to solve this selectivity challenge using traditional small-molecule inhibitors.

In recent years, Proteolysis-Targeting Chimeras (PROTACs) have emerged from the fringes of Chemical Biology to become powerful pharmacological tools and promising therapeutics^6^. PROTACs are bifunctional molecules that induce proximity between E3 ligase complexes and intracellular target proteins, resulting in ubiquitination and subsequent proteasomal degradation of the target protein. This exquisite reprogramming of the physiological cellular degradation machinery was first demonstrated by Crews, Deshaies and others over 20 years ago^7^, but it is only comparatively recently that the technology has matured to the point where it has entered mainstream drug discovery.

The unique mechanism of action of PROTACs imbues them with several potential advantages over traditional small-molecules, including targeting all protein function to replicate a knock-out phenotype, the ability to utilise non-functional binders, and the opportunity for increased selectivity arising from the requirement to form a productive ternary complex between the PROTAC, E3 ligase complex and the target protein. When a PROTAC induces proximity between an E3 ligase complex and its new target protein, new protein-protein interactions can form.

While many groups have sought to optimise formation of the ternary complex in order to increase degradation efficiency, this has led to mixed results^8^. In retrospect, this can be rationalised by the observation that PROTACs act as catalysts, and overstabilisation of any transient species in a catalytic cycle will not deliver maximal efficiency of that cycle^9^. Intriguingly, several reports of PROTACs incorporating non-selective kinase binders have demonstrated that degradation is only observed for a subset of targets to which the kinase binder portion of the PROTAC engages^10,11^. The kinases which are degraded are also dependent on the E3 ligase partner recruited by the PROTAC. While these reports represent examples of increased selectivity arising from the requirement to form a productive ternary complex, they are empirical observations and were not designed in a priori.

## Commentary

Now, two recent papers from Kofink et al. and Cantley et al. have prospectively targeted the PROTAC-mediated ternary complex to solve the apparently intractable SMARCA2/4 selectivity problem^12,13^.

It has been reported previously by Farnaby et al. that potent dual SMARCA2/4 degradation can be achieved using PROTACs derived from ligands targeting the druggable SMARCA2/4 bromodomains rather than the less tractable ATPase domains^14^. While inhibition of the bromodomains fails deliver antiproliferative effects, this is not an issue for a PROTAC where the entire protein is degraded. This ternary complex driven optimisation led to dual PROTAC ACBI1, which replicates the SMARCA2 RNAi phenotype in SMARCA4 deficient cell lines, but does not achieve degradation selectivity for SMARCA2.

Kofink et al. build on the identification of ACBI1, with the challenging task of achieving both selective SMARCA2 degradation and oral bioavailability^12^. This started with careful selection of the SMARCA2/4 bromodomain binder to minimise hydrogen bond donors and increase rigidity, which has been shown to improve likelihood of increased oral bioavailability for compounds beyond the rule-of-five^15^.

**Fig. 1:**
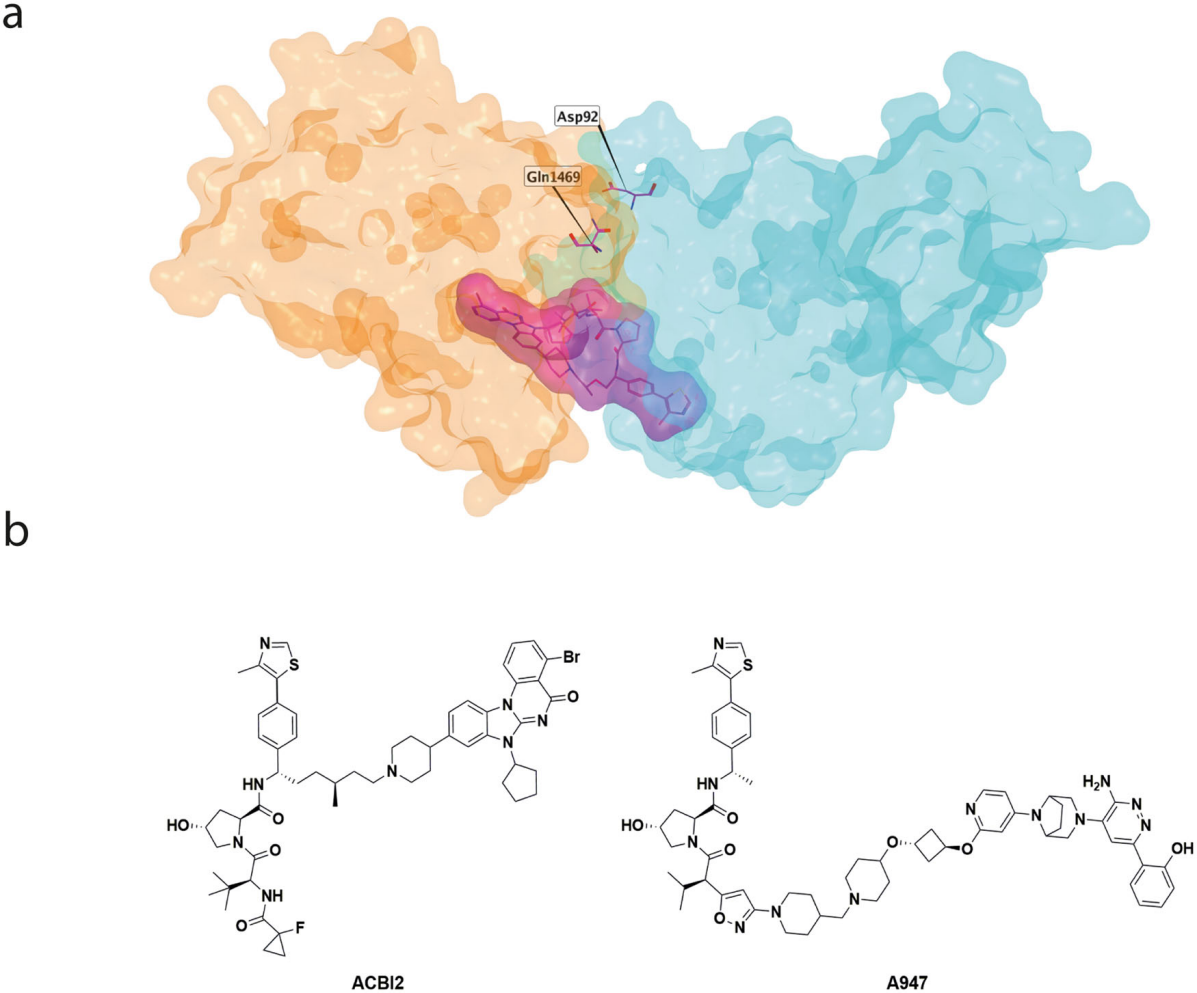
PROTAC-mediated ternary complex formation between SMARCA2 and the E3 ligase, VHL. **a** A representation of a SMARCA2-PROTAC-VHL ternary complex indicating SMARCA2-exclusive PROTAC-induced interactions (based on PDB: 7Z76, SMARCA2, =orange; PROTAC = purple; VHL = blue). **b** Chemical structures of SMARCA2-selective VHL PROTACs ACBI2 (left) and A947 (right).

A key aspect of this work was the availability of ternary complex crystal structures which highlighted the proximity of the SMARCA2 bromodomain and VHL binding sites in the complex, indicating that only short linkers would be necessary. This linker exploration identified extraordinarily selective SMARCA2 degraders with the most selective exhibiting >1000-fold degradation selectivity over SMARCA4. This could be rationalised by a ternary crystal structure of a close analogue, indicating a PROTAC-induced protein-protein interaction with SMARCA2 residue Gln1469 which is not conserved in SMARCA4 (Fig. 1a). Unfortunately, the properties of the highly SMARCA2-selective compounds did not have appropriate physicochemical properties to achieve oral exposure. This emphasises the compromises that must be made in the multi-parameter optimisation of therapeutic PROTACs, as the precise molecular conformation required to achieve ternary complex selectivity may not overlap with the restricted conformations required to achieve oral bioavailability.

Further work exploring extension of the linker between the SMARCA2/4 and VHL binders began to introduce the desired oral bioavailability into the PROTACs, at the expense of some SMARCA2 selectivity. Ultimately this led to the discovery of PROTAC ACBI2 which possess nanomolar degradation potency for SMARCA2, ~30-fold degradation selectivity over SMARCA4 and oral bioavailability of 22% in mouse (Fig. 1b). When ACBI2 was dosed orally in a SMARCA2-sensitive mouse xenograft model, almost complete degradation of SMARCA2 was achieved although this was associated with tumour stasis rather than the hoped-for regression.

The second paper from Cantley et al. describes a structurally distinct and highly potent SMARCA2-selective PROTAC A947 (Fig. 1b)^13^. In this case, an alternative exit vector from the VHL E3 ligase binder was used, connected to a more advanced SMARCA2/4 ligand, similar to that used previously in ACBI1^14^. This resulted in extremely potent, sub-nanomolar degradation of SMARCA2 with ~30x selectivity over SMARCA4. In this case, the physicochemical properties of the SMARCA2/4 ligand used in A947 proved to be restrictive to IV dosing.

When A947 was administered in SMARCA4-mutant xenograft studies, tumour stasis was again observed despite >95% reduction of SMARCA2 levels in the tumours. Interestingly, it was found that selective degradation of SMARCA2 could be achieved with partial sparing of SMARCA4, consistent with the selectivity profile observed in vitro, indicating the potential for SMARCA2 degradation selectivity by appropriate dose selection. Given SMARCA2 degradation failed to meet expectations for tumour efficacy, combinations were explored, which led to identification of in vitro sensitisation to apoptosis through combination with MCL1 inhibitors.

## Conclusion

Both papers demonstrate the unique benefit of PROTACs both in the utilisation of non-functionally relevant binding sites and critically for achieving selectivity through complementary ternary complex formation, delivering selective pharmacological SMARCA2 degradation. In both cases, the SMARCA2-SMARCA4 synthetic lethal relationship was validated in vivo and built on the existing in vitro evidence. Others looking to solve intractable selectivity challenges will no doubt be inspired by these results, and we can expect to see further examples of this strategy appearing in the literature in the future.
